# The Sanatory Club of Buffalo

**Published:** 1899-02

**Authors:** Thomas B. Carpenter

**Affiliations:** Secretary


### Society Proceedings.

### THE SANATORY CLUB OF BUFFALO.

REPORTED BY THOMAS B. CARPENTER, M. D., Secretary.

*Regular Meeting, December 14, 1898.*

THE meeting was called to order in the lecture room of the Buffalo Law School at 8.30 p. m., by the President, Dr. HENRY R. HOPKINS, lecturer on hygiene at the University of Buffalo, who introduced the topic of the evening with the following remarks :

It was the feeling of the topic committee in preparing this program that a double purpose should be served, not an unimportant part—though perhaps a secondary one—being to create an interest in the country at large in this very important subdivision of the subject of military hygiene. To the end that the matter might receive perhaps a little attention, a circular letter was issued by the committee and sent to the heads of the army, to various representative men and to the governors of states. As a result, a number of letters have been received, some of them of so much interest that I shall ask the secretary to read those which bear directly upon the general purpose of our meeting this evening.

The secretary then read letters of sympathy and approval from the State Board of Health of Michigan, Surgeon-General Sternberg of the Army, Brigadier-General Merriam, of California, Major-General Wm. Montrose Graham, Commanding Second Army Corps, from the Chief Signal Officer of the Army, from the Governor of Indiana and others.

### HYGIENIC CAMPS.

THE PRESIDENT—In what the chair has to say regarding the main purpose of our presence here this evening, *i. e.*, a discussion of the requisites of hygienic camps, he wishes to be exceedingly brief because we have a long program, and there is no necessity for being otherwise than concise.

It is the common knowledge of all informed men, that from the earliest times the fatality attending camp life is far in excess of that attending the march or line of battle. There has been no exception to this experience. If it were not that there has come into the minds of sanitarians within the last thirty years, something which is entirely new so far as sanitary science is concerned, there might possibly be criticism as to the expediency of discussing this question at this time, but within the last thirty years, since the Civil war, discoveries have been made as to the nature of the communicable diseases, discoveries which are of such an important character that the subject of the communication of these diseases needs to be restated and discussed anew, in so far as their prevention in camp is concerned. For this reason, prior to thirty years ago we had opinions as to how diphtheria was communicated, and in regard to the spread of dysentery, but we had nothing but opinions and our practice was of the vague and uncertain character which always attends action when that action is guided simply by opinion. But the labors of the microscopist and bacteriologist have replaced these opinions with more solid scientific material which produces in the mind *conviction* a very different affair. We now know positively how typhoid fever is communicated ; we know accurately how diphtheria is communicated, and for that reason it is proper that we should get together and discuss the question of hygienic camps.

Permit me a single moment as to the trend such discussion should take. It has seemed to the topic committee, and with that opinion I concur, that the time is past when the philosophy of the hygienic camp should be discussed and we do not propose spending time to discuss it. The time has come, in the opinion of your topic committee, when we should concentrate our efforts upon a discussion of the means of demonstrated value in civil life, which may be put into operation in military life to produce those conditions of health for which they have proved adequate over and over again in civil life. We are not to discuss philosophy, nor expediency, nor desirability; we have moved on beyond that, and have come together to discuss what approved appliances of civil life may be adapted to camp life, to the end that the same beneficial results, the prevention of disease, will prevail in the camp that have been known to follow under civil conditions, the manner by which our camps may have the simple but priceless advantage of clean soil, pure water, fresh air and therefore possibly be hygienic camps.

A single thought more, to which I will not commit the topic committee, but assume the entire responsibility myself—that is, that the disregard, which seems to appear in time of war, of the lives of the common soldier, has within it something of heathenism; that in America, where the common soldier is known to be a member of the nobility, where the muleteer and company cook are of the royal family of sovereigns, rulers of the land, it is exceedingly important, nay, imperative, that this sovereign of the people be protected in every phase of his life, particularly that life which he freely offers for the defense of the common country. Therefore, the sanitarian feels impelled, inasmuch as these lives are so important, to declare what may be done for their preservation, particularly as they are exposed in the camp of mobilisation or instruction.

The first paper is entitled, The camp of instruction, and will be presented by Lieutenant Harris, 13th Regiment, U. S. I. It is fortunate for us that he comes to the discussion of this matter fresh from our campaign of Santiago, and his knowledge is intimate and minute in regard to the matters of which he will speak.

Lieut. Peter C. Harris, Quartermaster 13th U. S. Infantry, then read a paper entitled, The camp of instruction. (See page 462.)

Major C. E. P. Babcock, 65th Regiment, N. Y. Vols., next read a paper on Camp sewers.

Mr. L. H. Knapp, C. E., Assistant Superintendent of the Buffalo Bureau of Water Supply, then presented the subject of Camp water supply.

Dr. Edward Clark made an appropriate presentation of the subject of Disposal of garbage and sewage.

Mr. H. C. Gardiner presented the question of Camp walks and streets.

Dr. M. A. Veeder, of Lyons, read the final paper of the evening, which was entitled, Relative importance of flies and water-supply in spreading disease.

These papers were then discussed by Captain ———— Norton, 65th N. Y. Vols.; Dr. W. D. Greene, Deputy Commissioner of Health; Major Eugene A. Smith, 65th N. Y. Vols.; Major A. H. Briggs, Surgeon 65th Regiment, N. Y. Vols.; Dr. William Warren Potter, Brevet Lieutenant Colonel, U. S. Vols.; Dr. A. L. Benedict and Mr. Dean Wilson, of the Health Department of Buffalo.

These papers and the discussions will be published in the Buffalo Medical Journal. A vote of thanks was tendered by the club to those who had participated in the interesting exercises of the evening, whereupon, at 11.15 p.m., the club adjourned.

